# Antiinflammatory effect of sevoflurane in open lung surgery with one-lung ventilation

**DOI:** 10.3325/cmj.2014.55.628

**Published:** 2014-12

**Authors:** Iztok Potočnik, Vesna Novak-Janković, Maja Šostarič, Ales Jerin, Tomaž Štupnik, Milan Skitek, Jasmina Markovič-Božič, Tomislav Klokočovnik

**Affiliations:** 1 Clinical Department of Anesthesiology and Intensive Therapy, University Medical Centre Ljubljana, Ljubljana, Slovenia; 2 Institute for Clinical Chemistry and Biochemistry, University Medical Centre Ljubljana, Ljubljana, Slovenia; 3 Clinical Department of Thoracic Surgery, University Medical Centre Ljubljana, Ljubljana, Slovenia; 4 Clinical Department of Cardiovascular Surgery, University Medical Centre Ljubljana, Ljubljana, Slovenia

## Abstract

**Aim:**

To prospectively assess the antiinflammatory effect of volatile anesthetic sevoflurane in patients undergoing open lung surgery with one lung ventilation (OLV).

**Methods:**

This prospective, randomized study included 40 patients undergoing thoracic surgery with OLV (NCT02188407). The patients were randomly allocated into two equal groups that received either propofol or sevoflurane. Four patients were excluded from the study because after surgery they received blood transfusion or non-steroid antiinflammatory drugs. Inflammatory mediators (interleukins 6, 8, and 10, C-reactive protein [CRP], and procalcitonin) were measured perioperatively. The infiltration of the nonoperated lung was assessed on chest x-rays and the oxygenation index was calculated. The major postoperative complications were counted.

**Results:**

Interleukin 6 levels were significantly higher in propofol than in sevoflurane group (*P* = 0.014). Preoperative CRP levels did not differ between the groups (*P* = 0.351) and in all patients they were lower than 20 mg/L, but postoperative CRP was significantly higher in propofol group (31 ± 6 vs 15 ± 7 ng/L; *P* = 0.035); Pre- and postoperative procalcitonin was within the reference range (<0.04 µg/L) in both groups. The oxygenation index was significantly lower in propofol group (339 ± 139 vs 465 ± 140; *P* = 0.021). There was no significant difference between the groups in lung infiltrates (*P* = 0.5849). The number of postoperative adverse events was higher in propofol group, but the difference was not-significant (5 vs 1; *P* = 0.115).

**Conclusion:**

The study suggests an antiinflammatory effect of sevoflurane in patients undergoing thoracotomy with OLV.

Acute lung injury (ALI) is the main complication of open lung surgery and is associated with a very high mortality rate (1-3). The incidence of ALI after lobectomy is 1%-7%. It is sometimes not easy to differentiate ALI from acute respiratory distress syndrome (ARDS) (4). The mortality rate in patients with ARDS and ALI is 72% and 33%, respectively (5,6).

In patients undergoing lung resection, mechanical ventilation and surgery may induce alveolar and systemic inflammatory responses (1,2,6). One-lung ventilation (OLV) has become a standard procedure for many interventions in thoracic surgery when there is a need to deflate the lung to facilitate the surgical procedure. It is the main cause of acute inflammatory response. The inflammatory reaction causes injury to the lung endothelium and induces the loss of endothelium integrity. This results in increased protein leak into the alveolar fluid and alveolar edema with ALI and ARDS (7-11).

A number of studies showed that sevoflurane attenuated cardiac ischemia-reperfusion injury (12). These results were lately transferred to lung surgery and it was found that volatile anesthetics had an impact on ischemic-reperfusion lung injury via local alveolar, but not the systemic antiinflammatory effects (13-15). Still, the long-lasting effects of anesthetics administered intraoperatively have not been evaluated. Demonstrating the influence of volatile anesthetics on the inflammatory response and the treatment outcome in patients undergoing open lung surgery with OLV is still a great challenge for thoracic anesthesiologists.

Although the effects of sevoflurane on release of inflammatory markers have been addressed before (13-15), the added value of our study is that we analyzed the systemic immunomodulatory effect of sevoflurane together with postoperative clinical outcomes and adverse effects. The following null hypothesis was tested: the administration of sevoflurane or propofol does not affect the systemic proinflammatory response after open lung surgery with OLV.

## Materials and methods

**Figure 1:**
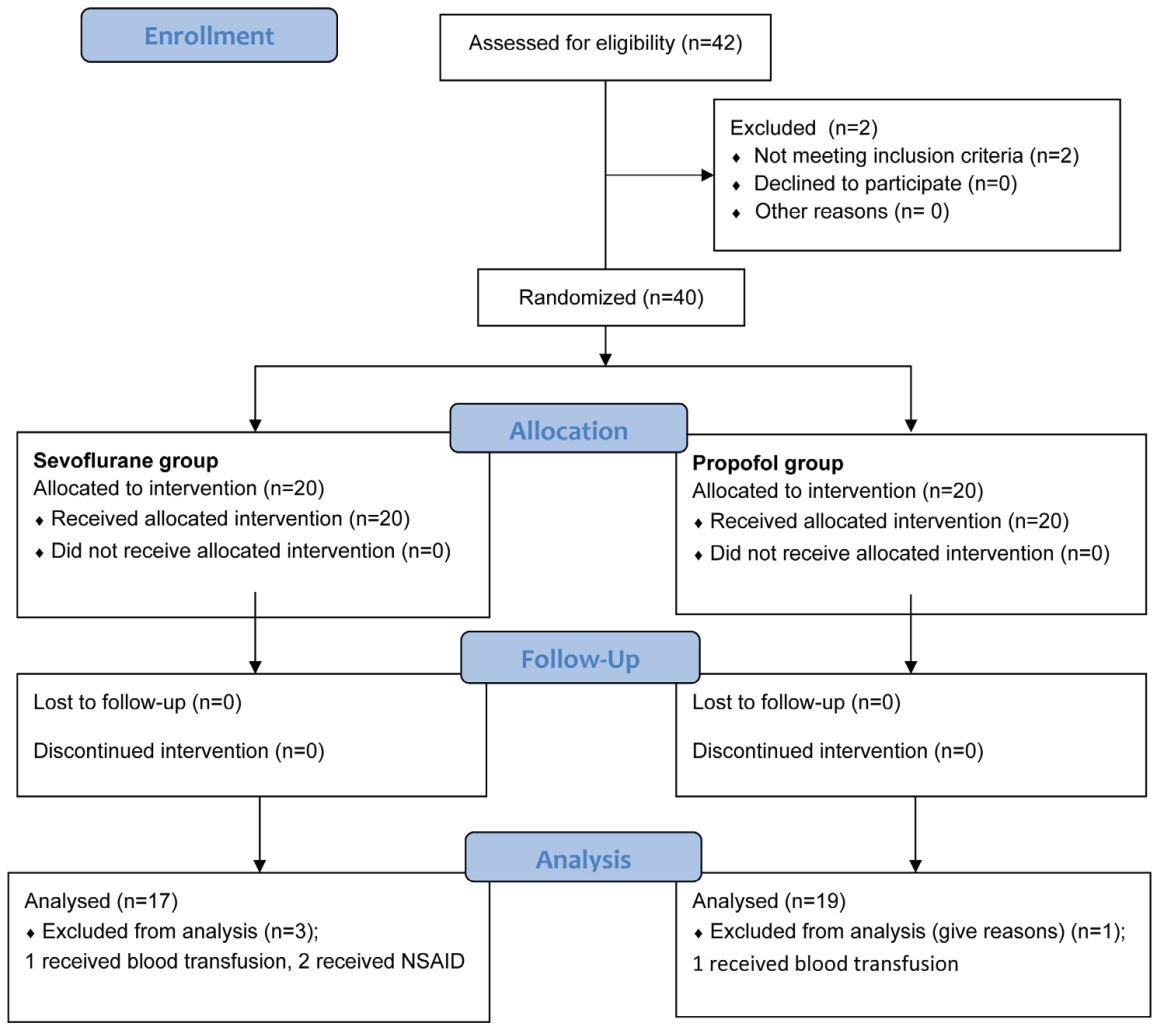
Flow diagram of the study.

This randomized, prospective study was conducted from 2009-2013 at the University Medical Centre Ljubljana, Department of Anesthesiology and Surgical Intensive Care and at the Department of Thoracic Surgery, in close cooperation with the Department of Clinical Chemistry and Biochemistry. The study was approved by the National Medical Ethics Committee of the Republic of Slovenia. It was registered in ClinicalTrials.gov under identifier: NCT02188407. Forty patients were enrolled: 20 in the propofol group and 20 in the sevoflurane group. Finally, 19 patients remained in propofol group (1 patient received intraoperative blood transfusion) and 17 in sevoflurane group (1 patient received intraoperative blood transfusion, 2 patients received non-steroid antiinflammatory drugs [NSAIDs] after surgery) (Figure 1).

The day before surgery the anesthesiologist informed the patients about the course and purpose of the study. All patients signed the informed consent. The patients included in the study were operated on by the same surgeon and anesthetized by the same anesthesiologist.

### Inclusion criteria

The study included patients aged 20-70 years with the American Society of Anesthesiologists (ASA) physical status I-III, scheduled for elective open lobectomy with OLV.

### Exclusion criteria

The exclusion criteria were as follows: history of drug hypersensitivity, drug addiction, treatment with psychotropic drugs, severe psychiatric and central nerve system diseases, persistent tobacco abuse, autoimmune system diseases, diabetes mellitus, cardiac failure (New York Heart Association class greater than 2), clinically relevant obstructive and restrictive lung diseases (vital capacity or forced expiratory volume in 1 s lower than 50% of the predicted values), pulmonary hypertension (mean pulmonary arterial pressure greater than 25 mm Hg), pre-existing coagulation disorders, and history of treatment with immunosuppressant drugs in the 4 weeks before surgery. Patients with evidence of pulmonary or systemic infections (CRP serum concentration greater than 5 mg/L, leucocytosis greater than 10.0 gigaparticles/L or body temperature greater than 37°C) were also excluded, as well as the patients who had received perioperative blood derivatives, steroids, or NSAIDs.

### Anesthesia regimen

All patients were given oral diazepam 5-10 mg one hour before surgery. On arrival to the operating room, they were randomly assigned to either propofol group or sevoflurane group. Randomization was performed using the random number generator. The surgeon and the anesthesiologist were blinded to the type of anesthesia.

Antibiotic prophylaxis with intravenous (iv) cefazoline 2 g/100 mL 0.9% NaCl was used in all patients. Standardized clinical monitoring devices were attached to all patients prior to induction of anesthesia. For extended hemodynamic monitoring, lithium dilution cardiac output system (LIDCO) was used. A thoracic epidural catheter was inserted at the T6-7 level.

Anesthesia induction in propofol group was performed with propofol (1.5-2.0 mg/kg) and in sevoflurane group with sevoflurane (deep breath with 6 V%). Before intubation all patients received remifentanil (0.5 mg/kg) and vecuronium (0.1 mg/kg). Anesthesia was maintained with propofol in propofol group (4-6 mg/kg/h) and with sevoflurane (V% 2-2.5) in sevoflurane group. The rate of remifentanil iv infusion was 0.3-0.5 µg/kg/min in both groups. The depth of anesthesia was measured by a bispectral index (BIS) monitor; BIS values were maintained at 40-60.

All patients were intubated with a left-sided double-lumen endobronchial tube and ventilated by volume-controlled ventilation, provided by a closed-circuit anesthesia ventilator. The tidal volume was set to 6 mL/kg. The peak inspiratory pressure was limited to 25 cm H_2_O. The fraction of inspired oxygen was adjusted to maintain oxyhemoglobin saturation at greater than 96% (fraction of inspired oxygen 0.3 to 0.4 before OLV; fraction of inspired oxygen 0.6 to 0.7 during OLV, and the respiratory rate to keep the Paco_2_ between 3.8-4.5 kPa). The positive end expiratory pressure (PEEP) was set to 5 cm H_2_O. Gas concentration and airway pressures were measured at the proximal end of the tube using ventilator-integrated functions. During OLV the tidal volume was set to 4 mL/kg and peak inspiratory pressure limited to 25 cm H_2_O. Other ventilation settings were maintained and PEEP was reduced to 3 cm H_2_O.

For hemodynamic management, the following algorithm was used: continuous infusion of 0.9% NaCl 6 mL/kg for the first hour, followed by 2.5 mL/kg/h. If oxygen delivery index (Do_2_I)<600 mL/min/m^2^, systemic vascular resistance (SVR)>800, and stroke volume variation (SVV)>10%, then 6% hydroxyethyl starch (HES) was infused until SVV decreased under 10%. If there was no improvement after 250 mL 6% HES-a, then dobutamine 1-10 µg/kg/min iv was introduced. If SVR<800 dyn · s/cm^5^ then ephedrine 5-10 mg iv was given. If the mean arterial pressure increased by more than 30% and the heart rate by more than 30% from baseline, then the infusion of remifentanil was increased by 0.1 µg/kg/min.

At the end of the procedure, the action of muscle relaxants was reversed with neostigmine 2.5 mg and atropine 1 mg iv. All patients were extubated in the operating theater and transferred to the recovery room.

### Postoperative management

After surgery, the patients stayed in the recovery room for one hour and then they were transferred to the intensive care unit of the Department of Thoracic Surgery. Standard postoperative monitoring generally used in these procedures was used. Hemodynamic monitoring using the LIDCO was continued after surgery. Oxygen titrated to the lowest level needed to achieve the target arterial oxygen saturation of 96% was administered via Venturi mask.

For postoperative hemodynamic management, the following algorithm was used: continuous infusion of 0.9% NaCl 1 mL/kg/h; if Do_2_I was less than 600 mL/min/m^2^ and SVR was higher than 800 dyn · s/cm^5^ then 6% hydroxyethyl starch was infused, if no improvement occurred after 250 mL 6% HES, then dobutamine 1-10 µg/kg/min was initiated, if SVR<800 then ephedrine 5-10 mg iv was given.

In the recovery room, all patients received 10 mL of 0.5% levobupivacaine and the level of the epidural sensory block was tested. Postoperative analgesia was administered by infusion of 0.25% levobupivacaine via an epidural catheter using a patient-controlled analgesia pump. The infusion rate was 5 mL/h and lockout time 30 min. Hourly pain assessments using a visual analogue scale (VAS) were performed. Patients with VAS scores greater than 3 received a rescue analgetic piritramide 3 mg intravenously.

### Measurements

We recorded demographic characteristics, time of surgery, time of OLV, and Do_2_I. Arterial blood samples for the determinations of cytokines (interleukins 6 [IL6], 8 [IL8], and 10 [IL10]) were drawn at the 5 time points: before induction, five minutes after the placement of the retractor, 10 minutes after the beginning of OLV, at the end of surgery, and 6 hours after surgery. CRP was measured preoperatively and 24 hours after the operation. For the analysis of serum CRP, IL6, IL8, and IL10, blood samples were collected without additive. After centrifugation, serum samples were stored at -20°C until analysis. Samples were analyzed in one batch. CRP, IL6, IL8, and IL10 were measured by a chemiluminescent immunometric assay; a high-sensitivity method with a detection limit of 0.3 mg/L was used for measuring CRP. Procalcitonin (PCT) levels were measured preoperatively, and 6 and 24 hours after the operation.

ALI and ARDS were both defined as the acute onset of bilateral infiltrates consistent with lung edema (bilateral pulmonary infiltrates on chest x-ray, pulmonary capillary wedge pressure lower than 18 mm Hg, the oxygenation index [ratio of partial arterial oxygen pressure to inspiratory fraction of oxygen = Pao_2_/FiO2] lower than 300 mm Hg for ALI, and lower than 200 mm Hg for ARDS) (16). Chest x-rays were taken 6 hours after the procedure to evaluate the infiltration of the nonoperated lung. The following scoring was used: 0 = no infiltration; 1 = partly infiltrated; and 2 = fully infiltrated. The oxygenation index was calculated 6 hours after the procedure.

### Secondary endpoints

The secondary endpoint was clinical postoperative outcome. The following major complications were determined postoperatively: diagnosed pneumonia, sepsis, ARDS, and death. These complications were classified as infectious complications (pneumonia, sepsis) and non-infectious complications (systemic inflammatory response syndrome [SIRS], ARDS).

### Statistical analysis

Statistical analysis was performed using SPSS (IBM, Armonk, NY, USA) 13.0 software package. Based on authors’ previous pilot study on 6 patients, to detect the significant difference in IL6 concentration with a significance level of 0.05 and a power of 80%, it was enough to have 16 patients in each group for the primary test. To compensate for withdrawals, we included 20 patients per group.

Demographic and other patents’ characteristics were compared using two-tailed *t* test with unequal variances, and differences in ASA, sex, lung infiltration, and postoperative complications using χ^2^ test. Plasma CRP, IL6, IL8, and IL10 concentrations were compared using repeated measures ANOVA. Continuous variables are presented as mean and standard deviation or median and range, and categorical data as the count. A *P* value of less than 0.05 was considered statistically significant.

## Results

The study included 40 patients, 20 in the propofol group and 20 in the sevoflurane group. None of the patients had signs of preoperative infection. All patients underwent open lung surgery because of the pulmonary cancer. Four patients were excluded because they required treatment with blood derivatives or NSAIDs. This left 19 patients in propofol group (1 patient received intraoperative blood transfusion) and 17 in sevoflurane group (1 patient received intraoperative blood transfusion, 2 patients received NSAID after surgery). There were no significant differences in patient characteristics between the groups (Table 1).

**Table 1:** Characteristics of patients who underwent open lung surgery with one lung ventilation (OLV) allocated into propofol or sevoflurane group*

	Sevoflurane group (N = 17)	Propofol group (N = 19)	*P* (two-tailed *t* test)
Age (years)	52.7 ± 14.6	60.9 ± 9.4	0.161
Weight (kg)	77.6 ± 13.5	81.9 ± 15.2	0.493
Height (cm)	174.3 ± 9.5	172.8 ± 9.5	0.731
Sex (f/m) (n)	8/9	10/9	
American Society of Anesthesiologists status 1/2/3 (n)	0/8/9	0/9/10	

*The results are presented as mean ± standard deviation or number of patients.

**Figure 2:**
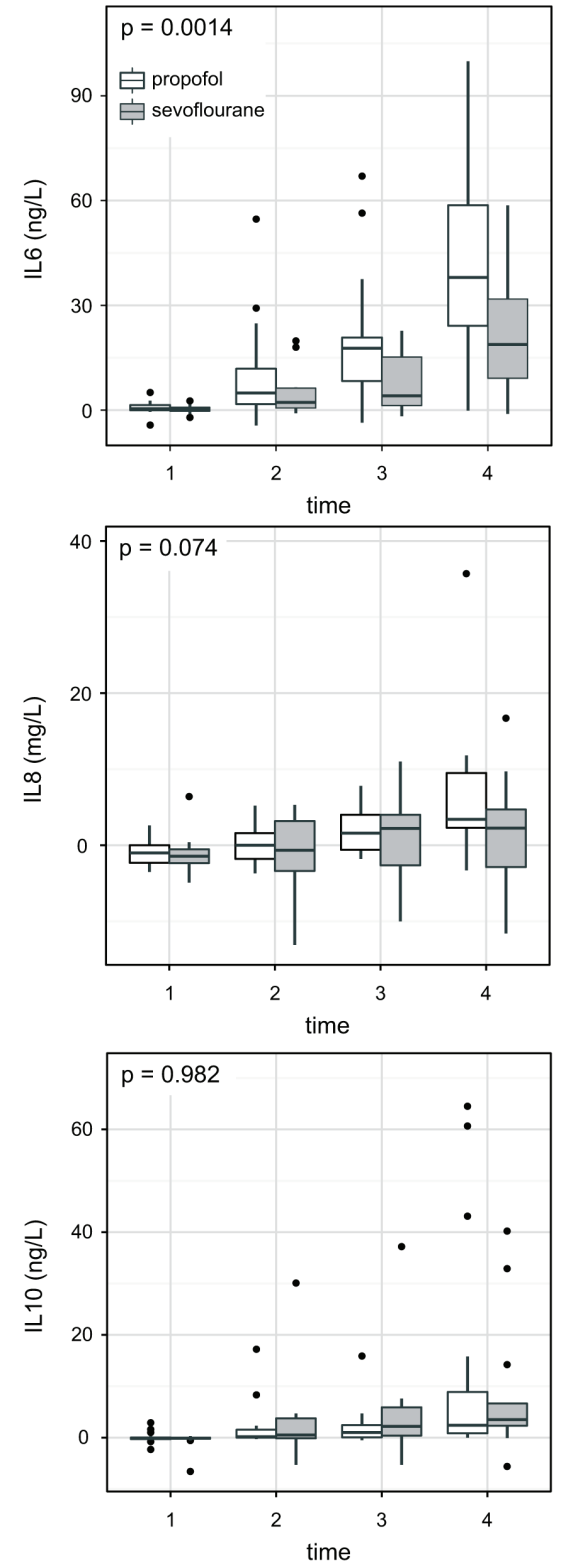
Interleukin 6 (IL 6), Interleukin 8 (IL 8), and Interleukin 10 (IL 10) levels. Time points: 1. Insertion of the retractor, 2. Beginning of one-lung ventilation, 3. End of surgery, 4. Six hours after surgery.

**Figure 3:**
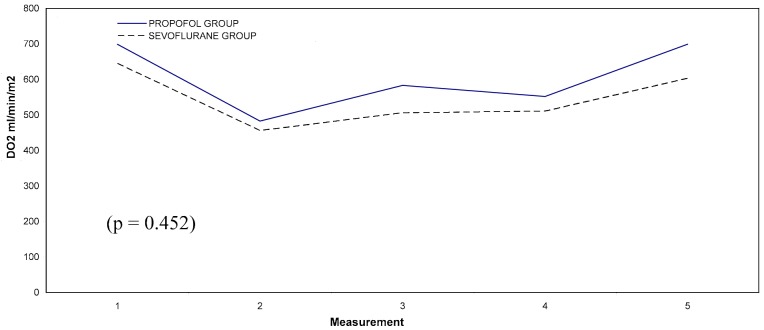
Oxygen delivery index (Do_2_I) (mL/min/m^2^). Time points: 1. Before induction, 2. After insertion of the retractor, 3. After the start of one-lung ventilation, 4. At the end of surgery, 5. Six hours after surgery. Repeated-measures ANOVA.

There were also no significant differences between the groups in intraoperative and postoperative variables that could have influenced the inflammatory response (Table 2). After OLV, both groups showed an increase in inflammatory mediators (calculated as the difference in concentrations of inflammatory mediators in plasma during and after the surgery) (Figure 2). However, the increase in proinflammatory mediators (IL6, IL8) was higher in propofol group. For IL6 the difference was significant (*P* = 0.014). The increase in IL10 was higher in sevoflurane group, but not significantly (Figure 3). The levels determined before the procedure were considered as baseline data; changes between baseline and later time points were analyzed using a repeated-measures ANOVA. There were no significant differences between the groups in oxygen consumption (*P* = 0.452) (Figure 2) and the infiltration of the nonoperated lung on a chest x-ray taken six hours after the surgery (*P* = 0.584) (Table 2). The postoperative oxygenation index (pO2/FiO2) was significantly higher in sevoflurane group (*P* = 0.021) (Table 2). Propofol group had more pulmonary infiltrates than sevoflurane group, but the difference was not significant (Table 3).

**Table 2:** Intraoperative and postoperative variables of patients who underwent open lung surgery with one lung ventilation (OLV) allocated into propofol or sevoflurane group*^†^

	Sevoflurane group (N = 17)	Propofol group (N = 19)	*P* (two-tailed *t* test)
Duration of operation (min)	139.3 ± 55	142.3 ± 47	0.893
Duration of OLV (min)	79.0 ± 10.3	82.5 ± 12.4	0.855
Right-sided thoracotomy (n)	12	15	
Number of blocked sympathetic segments (n)	8.2 ± 1.5	8.6 ± 1.4	0.831
Intraoperative consumption of remifentanil/kg BW (mg)	39 ± 5	55 ± 4	0.192
Intraoperative blood loss (mL)	158 ± 102	200 ± 85	0.652
Perioperative 0.9% NaCL (mL)	595 ± 189	570 ± 250	0.833
Perioperative 6% HES (mL)	537 ± 185	378 ± 184	0.191
Perioperative ephedrine (mg)	7.2 ± 4.2	20.8 ± 8.3	0.101
Perioperative dobutamine (µg/kg/min)	0.03 ± 0.004	0.02 ± 0.003	0.891
Postoperative piritramide (mg)	3.4 ± 1.2	3.5 ± 1.7	0.882
Postoperative VAS	3.0 ± 2.1	3.4 ± 2.7	0.323
Postoperative pO2/FiO2 index (mm Hg)*	465 ± 168	339 ± 139	0.021

*Abbreviations: HES – hydroxyethyl starch; VAS – visual analogue score; BW – body weight.

†Results are expressed as mean ± standard deviation unless otherwise indicated.

**Table 3:** Estimated infiltration of the nonoperated lung on a chest x-ray taken six hours after surgery in patients who underwent open lung surgery with one lung ventilation (OLV) allocated into propofol or sevoflurane group

Status, n (%)	Sevoflurane group (n = 17)	Propofol group (n = 19)
Clear lungs	15 (88)	15 (79)
Partly infiltrated lungs	2 (12)	3 (16)
Totally infiltrated lungs	0	1 (5)

*The difference between the sevoflurane and propofol group was not significant (*P* = 0.584, χ^2^ test.)

CRP and PCT as additional parameters for inflammation were assessed pre- and post operatively. Preoperative CRP was within the reference range in both groups (<20 mg/L). 24 hours after the surgery it was significantly lower in sevoflurane group (15 ± 6 ng/mL) than in propofol group (31 ± 7 ng/mL; *P* = 0.035). Before and 6 hours after the surgery PCT in both groups was lower than 0.04 µg/L. The overall number of adverse events was higher in propofol group, but the difference was not significant (4 vs 1; *P* = 0.115) (Table 4).

**Table 4:** Postoperative major complications in patients who underwent open lung surgery with one lung ventilation (OLV) allocated into propofol or sevoflurane group*

	Number of events		
	Propofol group (n = 19)	Sevoflurane group (n = 17)	*P* (χ^2^ test)
Infectious inflammation (pneumonia)	3	1	0.343
Non-infectious inflammation (ARDS, SIRS)	2	0	0.275
Death	0	0	x
Total	5	1	<0.05

*Abbreviations: SIRS – systemic inflammatory response syndrome; ARDS – acute respiratory distress syndrome.

## Discussion

The principal finding of the current study is that sevoflurane suppresses the systemic inflammatory reaction after OLV in open lung surgery. Lung injury after thoracic surgery is relatively uncommon, but it constitutes a major complication of this treatment, associated with high mortality rates (2,3). Recent studies have shown a combined frequency of ALI and ARDS of 3.9% (9). There are several possible triggers for ALI development during OLV for thoracic surgery. During OLV, the operated lung remains temporarily completely atelectatic, and hypo-perfusion occurs due to hypoxic vasoconstriction. Lung reexpansion and tissue reperfusion might reflect an ischemia-reperfusion injury, which can explain the underlying mechanism of inflammation (1,2,17-19). Other reasons may be high inspiratory oxygen concentration, surgical trauma, and the type of lung ventilation (1,2,17).

Cell and tissue injury related to an inflammatory response is the result of a complex array of mediators released by activated phagocytes, such as neutrophils and macrophages, or target cells, such as activated alveolar epithelial cells (AEC). Cytokines and chemokines are implicated in the recruitment of effector cells toward target tissues. Tumor necrosis factor alpha (TNF-α) and interleukins are strong neutrophil chemoattractants (13).

The interest in cell protective properties of volatile anesthetics first appeared in cardiac surgery, as they were shown to have cardioprotective effects (12). In a model of AEC injury, volatile anesthetics altered the secretion of inflammatory mediators upon IL 1β stimulation (20). Halothane, isoflurane, and enflurane decreased the production of IL6, macrophage inflammatory protein-2, and MCP-1 protein (19). Also, in an in vitro model sevoflurane decreased the expression of chemokines and attenuated chemotaxis (21). Coexposing AECs to endotoxin and sevoflurane treatment attenuated the inflammatory response after the onset of injury (21).

De Conno et al (13) found that the increase in inflammatory alveolar mediators IL6, IL8, TNF-α, IL1β, and MCP-1 upon OLV for thoracic surgery was smaller in sevoflurane than propofol group, meaning that propofol group had a significantly more prominent inflammatory reaction (13). Schilling et al (15) also showed increased concentrations of alveolar proinflammatory mediators in the ventilated lung after OLV. Both desflurane and sevoflurane were found to suppress the local alveolar, but not the systemic inflammatory response to OLV and thoracic surgery (15).

De Conno et al (13) and Schilling (15) measured cytokine concentrations in bronchoalveolar lavage (BAL). Both of these studies reported that sevoflurane reduced alveolar cytokine concentrations (13,15). We, on the other hand, measured these concentrations in serum samples and were interested in the impact of sevoflurane on the systemic inflammatory response.

Schilling et al claimed that the major limitation of their study was a short postoperative observation period: cytokine concentrations were determined immediately upon intubation and at the end of surgery (15). In our study, cytokine concentrations were measured at several time points during surgery and at 6 hours postoperatively.

Like Schilling et al (15), we measured concentrations of the major proinflammatory cytokines IL6 and IL8, and antiinflammatory cytokine IL10. However, unlike De Conno et al and Schilling et al (13,15) we did not determine TNFα because of its very short half-life and difficulties of its detection.

Our results showed more elevated concentrations of proinflammatory cytokines (IL6, IL8) in propofol than in sevoflurane group, confirming the antiinflammatory effect of sevoflurane. Schilling et al found significant difference between the groups in alveolar IL6 concentrations, but not in other proinflammatory cytokines. Our study also found a more pronounced increase in IL8 concentrations in propofol than in sevoflurane group. We were able to detect the difference because IL8 is released more slowly than IL6 (22) and we had a longer monitoring period.

In contrast to Schilling et al, who showed no increase in alveolar and serum concentrations of IL10 in either group, our results demonstrated elevated IL10 levels in both groups (15). The increase was more pronounced in sevoflurane group but the difference was not significant. These findings provide more evidence for antiinflammatory effects of sevoflurane.

Inflammatory response is affected by the duration of surgery, mechanical ventilation, the OLV time, and surgical tissue trauma (23). In our study, these factors can be excluded because there was no difference in the type and duration of the procedure, and OLV length, and because the procedures were carried out in a blinded fashion by the same surgeon and anesthesiologist.

In order to rule out infection and other immunological abnormalities as a possible cause of the enhanced inflammatory response, CRP and PCT levels were determined peri- and post-operatively. Preoperatively CRP and PCT were within the reference range in both groups, with no significant difference between groups; postoperative PCT was within the reference range in both group, but CRP levels were significantly higher in propofol than in sevoflurane group. This again confirms the antiinflammatory effect of sevoflurane. Propofol group had a higher rate of postoperative pneumonia, which could be the reason for the postoperative inflammation. However, we could exclude pneumonia as a reason for higher cytokines levels during the operation and in early postoperative period, since PCT was within the reference range.

Enhanced inflammatory response can also be related to tissue hypoxia (24). However, normal and non-significantly different Do_2_I values in the two groups suggested that the inflammatory response was not a consequence of hemodynamic instability or lower tissue oxygen delivery during the procedure. The groups also did not differ in the volume of the received colloids, crystalloids, and vasoactive agents.

Unlike the studies by De Conno et al and Schilling et al (13,15), we performed lung damage assessment after the operation. Propofol group had more pulmonary infiltrates than sevoflurane group, but the difference was not significant. The infiltrates may be associated with cardiac failure, but ruling out this possibility requires pulmonary capillary wedge pressure measurements with insertion of a Swan-Ganz catheter. This method, however, is too invasive to be used in such procedures. Lung infiltrates may also be a result of fluid overload but the non-significant difference in the amount of received crystalloids and colloids between the two groups does not support this assumption.

ALI and ARDS can be differentiated by the degree of hypoxemia (16). The oxygenation index in patients with ALI is 200 to 300 mm Hg. Hypoxemia is more pronounced in patients with ARDS, and their oxygenation index is lower than 200 mm Hg. We did not find a high-degree lung injury in either group, but we did find a significantly higher oxygenation index in sevoflurane group. Hence we can conclude that a more extensive lung injury occurred in propofol group and that sevoflurane had a lung-protective role.

Pain is another factor enhancing systemic inflammatory response and increasing serum cytokine levels (25). In our study, possible impact of pain-related stress on the inflammatory response can be excluded. There was no significant difference between the groups in the amount of remifentanil received during surgery. Also, there was no significant difference in postoperative VAS scores. The number of blocked spinal segments was equal in both groups and there were no differences in requirements for additional analgesia with piritramide.

Blood transfusion can also cause acute lung injury (26). Since we excluded the patients who had received perioperative blood transfusion, this cannot be a factor causing perioperative inflammation.

The overall number of major postoperative complications in sevoflurane group was lower than in propofol group. The number of non-infectious complications that can be a result of stronger intraoperative inflammation was also higher in propofol group, suggesting an improved clinical outcome in sevoflurane group.

A major advantage of this investigation over other studies is that it assessed the systemic cytokine release and postoperative adverse events, as well as performed clinical testing. The observation period was extended to the postoperative period and the positive postoperative clinical influence of the sevoflurane was confirmed, since the sevoflurane group had a lower rate of postoperative complications. However, even longer observation period would be required to clarify possible long-lasting effects of intraoperative medications. The main limitations of the study are a relatively small sample, short duration, and a not detailed enough evaluation of the clinical outcome.

This prospective randomized clinical study demonstrated a possible antiinflammatory effect of the volatile anesthetic sevoflurane in patients undergoing open lung surgery with OLV. An additional important finding was that sevoflurane showed systemic antiinflammatory action with fewer major postoperative complications. Total intravenous anesthesia with propofol has so far been the golden standard for open lung surgery. This study suggests that volatile anesthetics might be used instead of it in this type of surgery.
